# Rheumatoid arthritis and myasthenia gravis: a case-based review of the therapeutic options

**DOI:** 10.1007/s10067-022-06062-w

**Published:** 2022-01-14

**Authors:** Riccardo Bixio, Davide Bertelle, Francesca Pistillo, Elisa Pedrollo, Antonio Carletto, Maurizio Rossini, Ombretta Viapiana

**Affiliations:** Rheumatology Section, Department of Medicine, University of Verona Hospital Trust, 37134 Verona, Italy

**Keywords:** Rheumatoid arthritis, Myasthenia gravis, bDMARDs, tsDMARDs, JAK inhibitors

## Abstract

**Introduction:**

Myasthenia gravis is an autoimmune disease affecting the neuromuscular junction, often associated with other autoimmune diseases, including rheumatoid arthritis. Patients with rheumatoid arthritis present an increased prevalence of myasthenia gravis compared to the general population. While these two diseases share some therapeutic options, such as glucocorticoids, methotrexate, and rituximab, there are no guidelines for treating concomitant disease. We aim to review the available evidence and to discuss the efficacy and safety of the therapeutic options in patients with rheumatoid arthritis associated with myasthenia gravis.

**Method:**

We described three patients with rheumatoid arthritis associated with myasthenia gravis and we performed a systematic review of the associated literature.

**Results:**

A 48-year-old man and two women (48 and 55 years old) with concomitant diagnoses of active rheumatoid arthritis and well-controlled myasthenia gravis are described. They were treated with methotrexate, leflunomide, upadacitinib, and adalimumab. None of them experienced changes in their myasthenic symptoms. We found 9 additional cases from our literature review. Methotrexate, rituximab, upadacitinib, diphenyl sulfone, auranofin, and loxoprofen sodium did not show an impact on the seven patients with previously well-controlled myasthenia. Glucocorticoids, methotrexate, and rituximab proved effective in active myasthenia gravis and arthritis. Conflicting data emerged for Tumor-necrosis factor inhibitors.

**Conclusions:**

Although the available evidence remains scarce, we consider glucocorticoids, methotrexate, and rituximab as safe and effective options. The role of tumor-necrosis factor inhibitors remains uncertain. Eventually, Janus Kinase inhibitors are a novel interesting option for these patients.

**Table:** 

**Key Points** • *To date, the only evidence on the treatment of patients with rheumatoid arthritis and concomitant myasthenia gravis derives from case reports.* • *Based on the review of the available case reports and on the cases we described, we consider glucocorticoids, methotrexate, and rituximab as safe and effective options, while the role of Tumor-necrosis factor inhibitors remains uncertain.* • *Based on the cases we described, Janus Kinase inhibitors are a novel interesting option for patients with concomitant rheumatoid arthritis and myasthenia gravis.*

## Introduction

Myasthenia gravis (MG) is a rare (prevalence of 150–250 cases per million) antibody-mediated autoimmune neuromuscular disorder, characterized by weakness of eye muscles alone (ocular MG) or alongside skeletal muscles (generalized MG). The disease pathogenesis has been well characterized and three main autoantibodies have been described: antibodies against the acetylcholine (ACh) receptor (AChR) (80%), antibodies against muscle-specific kinase (MuSK), and antibodies against lipoprotein-receptor-related protein 4 (LRP4) [1]. MG patients present an increased frequency of a second autoimmune disease (around 13–22%), the most common being autoimmune thyroiditis (10–12%), followed by systemic lupus erythematosus (1–8%) [2, 3]. Thymectomy was associated with an increased risk of developing a second autoimmune disease in patients with MG (*OR* 4.4) [3], suggesting a possible underlying defect in autoreactive clones deletion [4]. The frequency of rheumatoid arthritis (RA) in patients with MG is estimated between 1–4% [5, 6]. A meta-analysis estimated a 3% prevalence of associated conditions [7]. Small populations of patients with MG reported similar values [2, 8]. On the other hand, patients with RA present an increased prevalence of MG compared to the general population (84/100.000 versus 35.8/100.000) [9]. The therapeutic options in MG range from acetylcholine esterase inhibitors (AchEi) (e.g., pyridostigmine) to traditional immunosuppressive drugs (e.g., glucocorticoids (GC), azathioprine, cyclosporine, mycophenolate mofetil, methotrexate (MTX), cyclophosphamide, and tacrolimus), short term treatments for the acute disease management as intravenous immunoglobulins (IVIG), plasma exchange (PLEX), and novel biologic therapies (rituximab, eculizumab) [10, 11]. These drugs only partially overlap with the treatments approved for RA, some of them, as D-penycillamine [12] and some Tumor Necrosis Factor inhibitors (TNFi) [13] have been correlated to MG development.

In this paper, we aim to discuss the therapeutic options for rheumatologists treating patients with RA associated with MG. We focused on therapies approved for RA, performing a literature review of cases of patients with RA and MG, to assess the impact of antirheumatic treatment on MG. We also described three patients with RA and MG, two of them treated with upadacitinib, a Janus Kinase inhibitor (JAKi) approved for RA, [14] and one patient treated with adalimumab (ADA).

## Methods

**Fig. 1:**
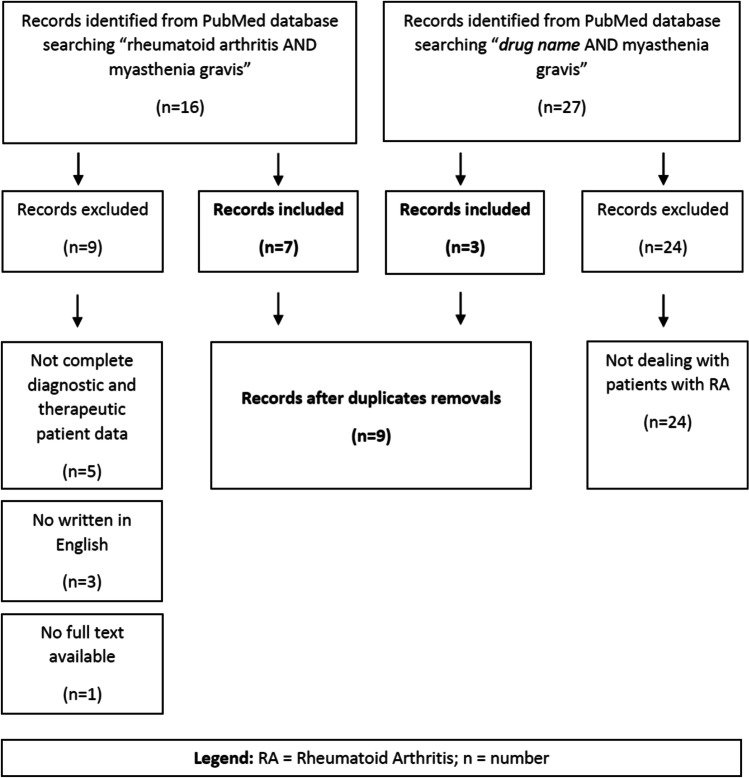
The article search flowchart

We described three patients’ clinical, laboratory, and therapeutic medical history based on their medical records. We collected the following data: gender, age, clinical course, C-reactive protein (CRP) level, Disease Activity Score-28 based on CRP (DAS28-CRP), pharmacological history, and outcome. Then, we performed a systematic review of the literature for therapeutic options in patients with MG and RA following the Preferred Reporting Items for Systematic Reviews and Meta-Analyses (PRISMA) guidelines. The inclusion criteria for articles were (1) article published from 1998 (approval date of infliximab, the first bDMARD used in rheumatology) [15] to October 2021, (2) article written in English, (3) full article available, and (4) complete diagnostic and therapeutic patient data available in the article. We explored the MEDLINE/Pubmed database on 1st of November 2021, using the following queries: (rheumatoid arthritis[Title]) AND (myasthenia gravis[Title]); (drug name[Title]) AND (myasthenia gravis[Title]). The article search flowchart is shown in Fig. 1, and the complete list of drug names searched for with correlated results are listed in Table 1. We screened the title and abstract of the retrieved work before inclusion to assess relevance. We checked the references of the retrieved articles to evaluate further reports. We checked and removed the duplicate results. Then, we read carefully through the full articles to include the reported cases in our review. All retrieved entries were independently reviewed by two different authors (RB and DB). We included a summary of their clinical and therapeutic history in Table 2. For classification of MG disease activity, severity, and response to therapy, we used the myasthenia gravis recommendations for clinical research standards of the Task Force of the Medical Scientific Advisory Board of the Myasthenia Gravis Foundation of America [16], taking into account the updates from the most recent International Consensus proposals [11]. For RA, we used the American College of Rheumatology and the European League against Rheumatism definitions [17, 18].

**Table 1:** Drugs name searching terms: list and results

	Retrieved papers	Patients with RA
Methotrexate	13	0
Leflunomide	4	0
Tumor necrosis factor inhibitors	1	1
Adalimumab	0	0
Certolizumab	1	1
Etanercept	0	0
Golimumab	0	1
Infliximab	0	0
Sarilumab	1	0
Tocilizumab	1	0
Abatacept	1	0
Janus Kinase Inhibitors	0	0
Baricitinib	0	0
Tofacitinib	0	0
Upadacitinib	0	0
Abatacept	1	0

*RA*, rheumatoid arthritis

**Table 2:** Demographical, clinical, and therapeutic characteristics of the patients with rheumatoid arthritis and myasthenia gravis

	Age (yrs)	Sex	Myasthenia gravis	Rheumatoid arthritis	Antirheumatic therapies	Clinical course
Oono et al., 2018 [20]	36	F	Generalized, thymectomy, seropositivity not specified, MM-2	Seropositivity not specified Remission	MTX, GC	No impact on MG was reported *Comorbidity:* rheumatoid meningitis (managed with GC increase)
Novella-Navarro et al., 2018 [21]	66	F	ocular, anti AchR + , new-onset	ACPA + , RF + Remission	MTX, ETN, RTX	MG onset after 6 weeks of ETN and 23 months of MTX. RTX (1000 mg, then 500 mg every 6 months) led to the remission of both MG and RA
Angelucci et al., 2010 [19]	68	F	Seropositivity not specified, PR	Seropositivity not specified Remission	GC, AZA, ADA	No impact on MG was reported. Exacerbation of MG after antirheumatic drugs withdrawal for pneumonia *Comorbidity:* Crohn’s disease, Uveitis
Horai et al., 2014 [22]	73	M	Manifestation not specified, seropositivity not specified, MM-2	ACPA + new-onset	MTX, GC	No impact on MG was reported
Kerkeni et al., 2008 [23]	50	F	Generalized, anti-AchR + , new-onset	Seronegative active	GC, MTX, AZA, IVIG, PEEX, RTX	New-onset of MG and active RA, therapy with AZA, IVIG, and PEEX ineffective; RTX (375 mg/m^2^ weekly over 4 weeks) led to remission of both MG and RA
Pelachas et al., 2020 [24]	42	F	Ocular, seronegative, new-onset	RF + , remission	MTX, ADA	MG onset after 18 months of ADA and 24 months of MTX
Fee et al., 2009 [25]	66	M	Generalized, anti-AchR + , new-onset	RF—no additional information	ETN	MG onset after 6 yrs of ETN, resolution after suspension
Wakata et al., 2001 [26]	56	F	Generalized, thymectomy, anti-AchR + , MM-0	RF + , new-onset	Diphenyl sulfone auranofin loxoprofen sodium	Resolution of RA, no impact on MG was reported
Zis et al., 2014	69	M	Generalized anti-AchR + , new-onset	ACPA + , new-onset	GC	Simultaneous onset of MG and RA, GC (75 mg/day) treatment led to remission of both
Described case 1	48	M	Generalized anti-AchR + , MM-2	ACPA + , RF + active	MTX, UPA	UPA and MTX led to remission of RA, with no impact on MG
Described case 2	55	F	Generalized anti-AchR + MM-2	ACPA + , RF- active	LEF, UPA	UPA and LEF led remission of RA, with no impact on MG
Described case 3	54	F	Generalized, anti-AchR + , MM-2	ACPA + , RF + active	GC, HCQ, CTZ-peg	CTZ-peg led to remission of RA, with no impact on MG

*ACPA*,anti-citrullinated peptide antibodies; *ADA*, adalimumab; *Anti-AchR*, anti-acetylcholine receptor antibodies; *AZA*, azathioprine; *CTZ-peg*, certolizumab pegol; *ETN*, etanercept; *FU*, follow-up; *GCs*, glucocorticosteroids; *HCQ*, hydroxychloroquine; *IVIG*, intravenous immunoglobulin; *LEF*, leflunomide; *MG*, myasthenia gravis; *MM-0*, no MG treatment received for at least 1 year; *MM-2*, minimal manifestations (only low dose cholinesterase inhibitors); *MTX*, methotrexate; *PEEX*, plasma-eritroexchange; *PR*, pharmacologic remission; *RA*, rheumatoid arthritis; *RF*, rheumatoid factor; *RM*, rheumatoid meningitis; *RTX*, rituximab; *UPA*, upadacitinib; *yrs*, years

## Results

### Our cases’ descriptions

#### Patient 1

A 48-year-old man was diagnosed with seropositive non-erosive RA (anti-citrullinated protein antibodies (ACPA) and rheumatoid factor (RF)) in 2012 after developing arthralgia and symmetrical and bilateral swelling of the small joints of the hands and of the wrists. He also presented a history of autoimmune thyroiditis. He received treatment with Methotrexate from 2012, initially at the dose of 15 mg/week, then reduced to 10 mg/week after showing gastrointestinal intolerance, while remaining in remission. In 2019 he developed generalized MG with palpebral ptosis and muscle weakness, with positive AChR antibodies (92 nmol/L) and negative anti-MusK antibodies. A computed tomography (CT) scan showed thymic hyperplasia, therefore thymectomy was performed later that year. MTX was temporarily halted. He received pyridostigmine (60 mg/three times daily (TID)), with minimal manifestations on AchEi (MM-2). In May 2020, there was a relapse of RA; MTX was resumed, but without relevant impact on disease activity. In April 2021, the patient DAS28-CRP was 4.82 (moderate disease activity), and it was therefore decided to begin treatment with Upadacitinib in association with MTX. After 1 month of follow-up, the patient reported a 60% improvement in pain and swelling (DAS28-CRP 2.74—low disease activity). After 4 months of follow-up, the patient RA was in remission (DAS28-CRP 1.65). During all this period, the patient did not require any change in the dose of pyridostigmine for MG.

#### Patient 2

A 55-year-old woman had a diagnosis of erosive seropositive (ACPA + , RF −) RA in 1991 with the involvement of wrists and hands joints. She was initially treated with sulfasalazine, then associated with MTX until January 2017. Because of gastrointestinal intolerance to MTX, she was then treated with leflunomide only, remaining in low disease activity. In 2008, she presented palpebral ptosis, diplopia, and generalized muscle weakness, leading to diagnosing MG. Thymectomy was performed in the same year, no immunosuppressive treatment was started, and she controlled her mild symptoms with pyridostigmine only (current dose 105 mg/day) (MM-2). In June 2021, she manifested an active RA (DAS28-CRP 3.45) confirmed by hand ultrasound, which showed active tenosynovitis at multiple locations. Given the disease activity and the erosive nature of her RA, Upadacitinib 15 mg/day was started. After 3 months of therapy with UPA, RA was in remission (DAS28-CRP 1.64), as confirmed by hand ultrasound, and the neurological examination did not show any change in MG.

#### Patient 3

A 48-year-old woman presented to our outpatient clinic in June 2015 with arthralgia of hands and feet. Her blood exams showed positivity of ACPA (117.9 CU), RF (38 KU/L), antinuclear antibodies (1:320), and anti-SSA antibodies. She had an increased erythrocyte sedimentation rate (44 mm/hour) and CRP (17 mg/L). Hand ultrasound showed active tenosynovitis without erosive damage. A diagnosis of RA was made, and she was initially treated with a low dose of GC (Prednisolone (PDN) 7.5 mg/day) and HCQ. In 2017, after a RA flare (CRP 15 mg/L), we decided to treat her with Certolizumab Pegol, associated with low doses of GC (PDN 2.5 mg/day). The patient had received a previous diagnosis of myasthenia gravis in 2000, with initial presentation of palpebral ptosis and diplopia, and positive anti-AchR antibodies (10 nmol/L). The MG was initially treated with high doses of GC and azathioprine, and after the initial remission, she was treated with Pyridostigmine only. In 2003, a CT scan revealed thymic hyperplasia and thymectomy was performed. An exacerbation of her MG occurred in 2008, and an IVIG cycle was administered with remission of the symptoms, controlled with a low dose of pyridostigmine (MM-2). From the diagnosis of RA to September 2021, her neurologic examinations did not show any changes in her MG disease activity (MM-2). At her last rheumatologic examination in October 2021, she was in remission (DAS28-CRP 1.99).

## Literature review

We retrieved 9 case reports of patients with concomitant RA and MG [19–27] and described three more cases. The clinical and therapeutic characteristics are summarized in Table 2. Six patients had active MG at a certain point of their history: three patients developed myasthenia gravis during treatment with TNFi (etanercept (ETN) *n* = 2, adalimumab (ADA) *n* = 1) alone or with MTX (2/3), one had an exacerbation of MG after the suspension of azathioprine and ADA for pneumonia, and one patient developed acute RA and MG at the same time. Between the six patients with active MG, two were successfully treated with RTX, one with GC only and one patient ameliorated MG symptoms after ETN suspension. For two patients, no details are provided. Nine patients were already affected by myasthenia gravis when the antirheumatic treatment was started. The following drugs did not show an impact on the seven patients with previously well-controlled MG (minimal manifestations with AchEi only (MM-2) *n* = 5, minimal manifestation without therapy (MM-0) *n* = 1, pharmacologic remission (PR) *n* = 1): methotrexate (*n* = 4), upadacitinib (*n* = 2), TNFi (ADA *n* = 1, Certolizumab pegol *n* = 1), diphenyl sulfone, auranofin, loxoprofen sodium (*n* = 1) (total greater than 7 because some patients were on combination therapy). There was great variability in follow-up ranging from a few days after the initial antirheumatic treatment to several years after its beginning. We did not retrieve any randomized controlled trial or meta-analysis or expert opinion article about our research topic.

## Discussion

Reports of patients with concomitant RA and MG are scarce in the literature, but additional information could be extrapolated by patients with MG treated with drugs approved as antirheumatic drugs. Methotrexate, the anchor drug for RA, although did not show efficacy on MG [28], is nonetheless suggested as a possible steroid-sparing agent in refractory MG [10], and our finding does not contraindicate its use. Our review illustrated two cases of rituximab effectiveness in concomitant RA and MG, coherently with existing recommendations for both diseases [10, 29]. We found conflicting evidence about TNFi: while some reports suggest etanercept could be effective in reducing MG symptoms in some patients [30], others associated the treatment with TNFi with symptoms worsening or MG development in patients treated with TNFi for RA and Psoriatic arthritis (PsA) [24, 25, 31, 32]. Tocilizumab, an interleukin-6 inhibitor, has been used with success in two patients with MG refractory to RTX [33]. Abatacept, a recombinant Cytotoxic T-Lymphocyte Antigen 4 (CTLA4) fusion molecule used in RA, has been reported as effective in a Nivolumab-induced MG in an oncologic patient [34]. Interestingly, the anti-CTLA 4 checkpoint inhibitor ipilimumab, used for melanomatous skin cancer, has been linked to MG development or worsening [35].

One of the novelties of our case reports is the utilization of JAKi (upadacitinib) in patients with pre-existent MG. To our knowledge, there is only one patient with active MG previously treated with another JAKi (ruxolitinib) for his myelodysplastic syndrome, with amelioration of both diseases [36]. Ruxolitinib, which is used to treat myelofibrosis, polycythemia vera, and graft versus host disease [37, 38], is not approved in Europe and the USA for RA. Alboini et al. reported that the patient’s acute MG went into remission after starting the treatment with ruxolitinib, even if the causal effect is difficult to assess since MG is a disease characterized by fluctuating disease activity and spontaneous remissions are possible [36]. Our patients had a well-controlled MG (MM-2) at the beginning of the new antirheumatic treatment; therefore, we could not bring further evidence about their efficacy on MG; however, we did not observe a worsening of their MG during follow-up. JAKi are known to reduce both effector T cells and B cell-mediated immunity, preserving T regulatory cell activity; furthermore, blocking the JAK-STAT pathway could reduce interferon signaling, which was overexpressed in patients with MG, particularly when associated with thymomas [39]. Therefore, this new class of drugs could be able to effectively modulate the different pathogenic mechanisms involved in MG [40–42]. Based on the previously exposed report and on the pathogenesis of the disease, we believe JAKi should be considered a safe and well-tolerated therapeutic option to treat patients with concomitant RA and MG, with a low risk of MG exacerbation or worsening. Our study is limited by its retrospective nature, being based on case reports from different centers and settings (rheumatologic, neurologic, internal medicine) characterized by a great degree of heterogeneity either in the neurological or rheumatological disease descriptions and by generally short follow-ups.

## Conclusions

According to our experience and the review of the literature, in patients with active RA and concomitant MG, we consider MTX a valid option as initial DMARD and confirm RTX efficacy as bDMARD. The TNFi role remains uncertain, but we would advise great caution about their use. Eventually, we believe that JAKi should be considered as a reasonable treatment option in such patients.

## Data Availability

PRIMA checklist is available as online additional file.
